# Intracerebroventricular Administration of Nerve Growth Factor Induces Gliogenesis in Sensory Ganglia, Dorsal Root, and within the Dorsal Root Entry Zone

**DOI:** 10.1155/2014/704259

**Published:** 2014-03-16

**Authors:** Johannes C. M. Schlachetzki, Donald P. Pizzo, Debbi A. Morrissette, Jürgen Winkler

**Affiliations:** ^1^Department of Molecular Neurology, Friedrich-Alexander-University Erlangen-Nürnberg (FAU), University Hospital Erlangen, Schwabachanlage 6, 91054 Erlangen, Germany; ^2^Department of Neurosciences, University of California, La Jolla, San Diego, CA 92093-9157, USA; ^3^Neurology Service, Veterans Affairs Medical Center, San Diego, CA 92161, USA

## Abstract

Previous studies indicated that intracerebroventricular administration of nerve growth factor (NGF) leads to massive Schwann cell hyperplasia surrounding the medulla oblongata and spinal cord. This study was designed to characterize the proliferation of peripheral glial cells, that is, Schwann and satellite cells, in the trigeminal ganglia and dorsal root ganglia (DRG) of adult rats during two weeks of NGF infusion using bromodeoxyuridine (BrdU) to label dividing cells. The trigeminal ganglia as well as the cervical and lumbar DRG were analyzed. Along the entire neuraxis a small number of dividing cells were observed within these regions under physiological condition. NGF infusion has dramatically increased the generation of new cells in the neuronal soma and axonal compartments of sensory ganglia and along the dorsal root and the dorsal root entry zone. Quantification of BrdU positive cells within sensory ganglia revealed a 2.3- to 3-fold increase in glial cells compared to controls with a similar response to NGF for the different peripheral ganglia examined. Immunofluorescent labeling with S100**β** revealed that Schwann and satellite cells underwent mitosis after NGF administration. These data indicate that intracerebroventricular NGF infusion significantly induces gliogenesis in trigeminal ganglia and the spinal sensory ganglia and along the dorsal root entry zone as well as the dorsal root.

## 1. Introduction

Nerve growth factor (NGF) is considered as one of the classical target-derived proteins essential for the development and maintenance of neural crest-derived sensory neurons in the peripheral nervous system (PNS) [1]. NGF has been shown to exert neuroprotective effects and even has been implicated in the treatment of chronic neurodegenerative diseases such as glaucoma and Alzheimer's disease [2, 3]. NGF mediates its effects in the PNS via two receptors: low-affinity p75 neurotrophin receptor (p75NTR) and high-affinity tyrosine kinase receptor A (TrkA). In the adult PNS, both receptors are expressed on neurons of trigeminal ganglia and dorsal root ganglia (DRG) [4–6]. With regards to the glial population in sensory ganglia, satellite cells express p75NTR and TrkA, while Schwann cells express p75NTR only [4, 7–11]. The NGF mediated activation of the nonselective p75NTR in the dorsal root ganglia (DRG) promotes axonal growth and migration of Schwann cells during development and adulthood as well as subsequent to injury [12–14]. It is also a characteristic marker for neural crest stem cells [15, 16].

Over the past decades it has been recognized that NGF plays an important role for cholinergic neurons of the basal forebrain (CBF) [17]. In particular, NGF has the ability to reverse deficits in numerous models of cholinergic deafferentiation [18–21]. These findings prompted the notion that NGF may be of therapeutic value for patients with Alzheimer's disease [22] since CBF neurons are severely affected during the course of this disease [23–25]. Due to the limited CNS bioavailability of peripherally administered NGF [26], several approaches to deliver exogenous NGF directly into the brain have been explored.

One widely used approach to deliver NGF to the CBF uses continuous intracerebroventricular (ICV) infusion [20, 27, 28]. ICV delivery of NGF results in a robust restoration of compromised cholinergic functions. In addition, due to the continuously present flow of cerebrospinal fluid (CSF), NGF diffusely spreads throughout the ventricular system and has access to numerous structures of the PNS. Since the DRG with its roots as well as the dorsal root entry zone (DREZ), the interface between the CNS and PNS, is immersed in CSF, ICV-administered NGF has access to these structures. Thereby, structural changes in the PNS were observed due to the distribution of NGF within the subarachnoid space. In particular, sprouting of sympathetic neurites, hypertrophy of sensory neurites [29–31], and Schwann cell hyperplasia [31–33] were noted in the adult rodent and primate. Neural crest stem cells (NCSC) give rise to all neuroglial cells of the PNS and after migration coalesce into ganglia consisting of the sensory neurons, satellite cells, and Schwann cells. Since the major cell populations within the adult DRG express NGF receptors they may respond to exogenous NGF application.

Thus, the present study aimed to detect more precisely the effect of NGF on distinct glial populations within the cervical and lumbar DRGs, trigeminal ganglia, dorsal root, and the DREZ with a particular focus on proliferation and phenotypic analysis.

## 2. Material and Methods

### 2.1. Animals

Male Fischer 344 albino rats (*n* = 9; Harlan Sprague-Dawley, Indianapolis, IN) weighed 281.8 ± 2.0 g (mean ± S.E.M.) at the start of the experiment. Animals were housed in pairs in plastic cages (48 × 27 × 20 cm) in a large, well-lit laboratory controlled for temperature (21°C) and maintained with a daily photoperiod of 12 hrs. Each animal had free access to water and was fed* ad libitum *on a standard laboratory diet (Teklad 4% Rat Diet 7001, Harlan Teklad, Madison, WI). All procedures were approved by the local animal care and use committee and performed at an institution fully accredited by the Association for the Assessment and Accreditation of Laboratory Animal Care International.

### 2.2. Surgical Procedure

Osmotic minipumps (Model 2002, Alza Co., Palo Alto, CA) were connected to cannulas (5 mm length; Plastics One Inc., Roanoke, VA) with vinyl tubing (size V/4; 3.5 cm length, Bolab Inc., Lake Havasu City, AZ). This infusion system was filled with human recombinant NGF (416.7 ng/*μ*L; a gift of Genentech; Palo Alto, CA) or vehicle (cytochrome c; 416.7 ng/*μ*L; Sigma Chemical Company, St. Louis, MO) dissolved in artificial cerebrospinal fluid (148 mM NaCl, 3 mM KCl, 1.4 mM CaCl_2_, 0.8 mM MgCl_2_, 1.5 mM Na_2_HPO_4_, and 0.2 mM NaH_2_PO_4_, at pH = 7.4) containing rat serum albumin (100 *μ*g/mL; Sigma) and gentamicin (50 *μ*g/mL; Sigma) resulting in a dose of 5 *μ*g/day.

Rats underwent surgery in a stereotaxic frame (David Kopf, Tujunga, CA) in a flat-skull orientation. Animals were anesthetized with an intramuscular injection consisting of 62.5 mg/kg ketamine (100 mg/mL; Fort Dodge Animal Health, Fort Dodge, IA), 3.175 mg/kg xylazine (100 mg/mL; Boehringer Ingelheim Vetmedica, St. Joseph, MO), and 0.625 mg/kg acepromazine maleate (10 mg/mL, Boehringer Ingelheim Vetmedica), diluted in 0.9% sterile saline. NGF or cytochrome c was infused into the lateral ventricle via the cannulas at AP +8.5 and lateral −1.5 taken from the interaural line according to the stereotaxic atlas of Paxinos and Watson [35]. A subcutaneous pocket in the midscapular area was created for placement of the osmotic pump. The number of animals in each group is as follows: untreated (*n* = 3), vehicle (*n* = 3), and NGF (*n* = 3). All animals were weighed daily throughout the experiment.

### 2.3. Labeling of Proliferating Cells and Tissue Processing

To label dividing cells all animals received one intraperitoneal injection of bromodeoxyuridine (BrdU; 50 mg/kg; Sigma, St. Louis, MO) each day for 14 d. On day 14, all animals were deeply anesthetized and perfused intracardially with 200 mL of 100 mM phosphate buffer (PB; pH 7.4) followed by 500 mL of ice-cold 4% paraformaldehyde in 100 mM PB. The trigeminal ganglia were removed and postfixed for 24 hrs in 4% paraformaldehyde. The spinal column was dissected and postfixed identically, and all samples were transferred to 0.32 M sucrose for cryoprotection. The entire spinal cord, in continuity with the dorsal roots and the DRGs, as well as the spinal nerves, was dissected from the spinal column. Individual cervical (C5–C7) and lumbar (L2–L4) DRGs attached to their corresponding spinal cord segments as well as the trigeminal ganglia were coronally sliced and embedded in a cryomold (Fisher Scientific, Pittsburgh, PA) using O.C.T. mounting medium (Tissue Tek, Torrance, CA). After rapidly freezing in liquid nitrogen-cooled isopentane to preserve tissue integrity, the molds were stored at −80°C until sectioned coronally on a cryostat (Cryo-Star HM 560V; Microm Instruments, Walldorf, Germany) at 40 *μ*m. Every section from a coronal series of the spinal ganglia was sequentially mounted across 10 Superfrost slides (Fisher Scientific) such that adjacent sections on each slide were 400 *μ*m apart. Slides were dried overnight at room temperature, stored at −80°C, and selected for both BrdU immunohistochemistry and immunofluorescence labeling.

To control for appropriate ICV positioning of the cannula, the entire brain was dissected. The forebrain at the level of the lateral ventricles was sliced coronally at a thickness of 40 *μ*m. Nissl staining was performed to visualize the cannula tract.

### 2.4. Immunohistochemistry

For the quantification of BrdU positive cells, one in two cryostat section series of the ganglia was stained using diaminobenzadine (DAB) immunohistochemistry. After two rinses in 0.1 M Tris-buffered saline (TBS; pH 7.5) for 15 min each, endogenous peroxidase activity was blocked by incubation in 0.6% H_2_O_2_ in TBS for 30 min followed by three TBS rinses for 15 min each. Subsequently, sections were treated with 50% formamide in 2X SSC (0.3 M NaCl, 0.03 sodium citrate) for 2 hrs at 65°C, rinsed for 15 min in 2X SSC, incubated for 30 min in 2N HCl at 37°C, and rinsed for 10 min in 0.1 M boric acid (pH 8.5). Several TBS rinses were followed by incubation in TBS/0.1% Triton X-100/5% normal donkey serum (TBS++) for 30 min. The sections were incubated overnight using a monoclonal anti-BrdU (1 : 100; Chemicon, Temecula, CA) in TBS++ at 4°C. After several rinses in TBS++ on the following day, sections were incubated for 2 hrs with donkey anti-mouse IgG biotin (1 : 250; Accurate Chemicals, Westbury, NY) diluted in TBS++ at room temperature. Sections were rinsed three times in TBS both before and after 1 hr incubation with avidin-biotin complex (ABC-Elite; Vector Laboratories, Burlingham, CA). BrdU labeling was visualized using DAB (0.025% mg DAB, 0.01% H_2_O_2_, and 0.6% NiCl_2_ in TBS) and terminated by rinsing the slides three times in TBS. Slides were dehydrated through an ascending series of alcohol and coverslipped with DPX (BDH Laboratory Supplies, Poole, England).

### 2.5. Quantification of Proliferating Cells

The counting procedures were performed on a light/fluorescence microscope (Olympus BH-2, Melville, NY). The sensory ganglia consist of two anatomically distinct compartments: a component predominantly containing axonal fibers, termed* axonal compartment*, and a second with ganglionic cell bodies, termed* neuronal soma compartment*. For the cervical ganglia, the neuronal soma compartment was outlined and measured in mm^2^. Subsequently, BrdU positive cells within the neuronal soma compartment were exhaustively counted in coronal sections from three cervical DRGs (C5, C6, and C7) of each animal. Cell counts were recorded using Stereo Investigator Software 4.05 (MicroBrightfield, Colchester, VT). For the quantification of BrdU positive cells of the trigeminal and lumbar ganglia, we used a systematic random counting procedure [36], similar to the optical dissector using a semiautomatic stereology system (StereoInvestigator, MicroBrightField, Williston, VT, USA). BrdU positive cells (through a 40 *μ*m axial distance) intersecting the uppermost focal plane (exclusion plane) and those intersecting the exclusion boundaries were not counted.

### 2.6. Immunofluorescence

Sections were processed for DNA denaturation as detailed above followed by several TBS rinses and incubation in TBS++ for 30 min. Primary antibodies were used as follows: sheep anti-BrdU (1 : 800; Biodesign, Saco, ME), rabbit anti-S100*β* (1 : 500, Swant, Bellinzona, Switzerland), and rabbit anti-NeuN (neuronal nuclei; 1 : 500, Millipore, Billerica, MA). Sections were incubated with primary antibodies for 48 hrs at +4°C. After several rinses in TBS++ for 10 min at room temperature, the secondary antibodies coupled to fluorescein isothiocyanate (FITC), cyanine 3 (CY3), and cyanine 5 (CY5) were applied for 2 hrs (all used at 1 : 250 in TBS++; Jackson ImmunoResearch, West Grove, PA). Sections were washed three times in TBS and coverslipped in 20% polyvinyl alcohol (average MW 30,000–70,000) in 50% glycerol (w/v) containing 2.5% w/v 1,4-diazabicyclo-[2.2.2]-octane (Sigma).

### 2.7. Statistical Analysis

Differences among experimental groups were evaluated by one-way ANOVA followed by* post hoc* Bonferroni's multiple comparison test to determine individual group differences with a criterion for significance set at *P* < 0.05.

## 3. Results

**Figure 1:**
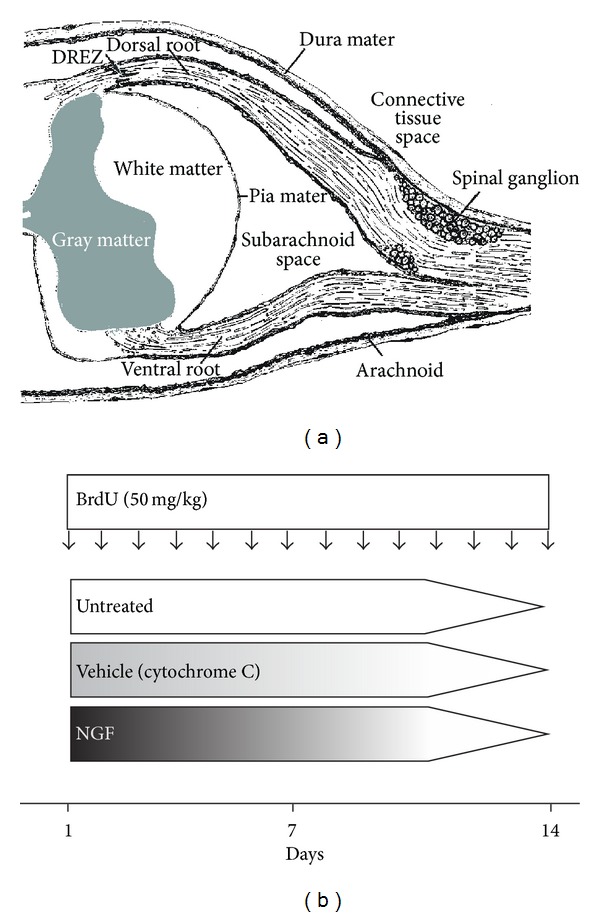
Cross section of the spinal cord and the PNS ((a) Cartoon adapted from Dyck and Thomas, 2005 [34]). Experimental design: vehicle (cytochrome c) or NGF was continuously delivered by ICV administration for 14 days. The untreated group did not undergo surgery. Intraperitoneal BrdU injections were given daily. *N* = 3 per group (b).

Aim of the present study was to characterize the NGF associated mitogenic effect on glial cells within the PNS, namely, the DREZ, dorsal root, and spinal ganglia (Figure 1(a)). For this aim, NGF was continuously delivered into the lateral ventricle for 14 days via an osmotic minipump (Figure 1(b)). Two weeks after pump implantation, rats of the NGF group weighed 244.8 ± 3.8 mg and rats of the vehicle control weighed 282.3 ± 5.7 mg (*P* < 0.05). Thus, ICV administered NGF resulted in significant lower weight compared to controls within the observed time period. It has been shown that ICV administration of NGF induces hypophagia [37]. This NGF-related effect is dose-dependent, centrally mediated, and reversible [37]. In particular, the NGF-mediated hypophagia has been suggested to be related to decreased hypothalamic cholecystokinin levels [38].

### 3.1. NGF Induced Cell Proliferation in the PNS

**Figure 2:**
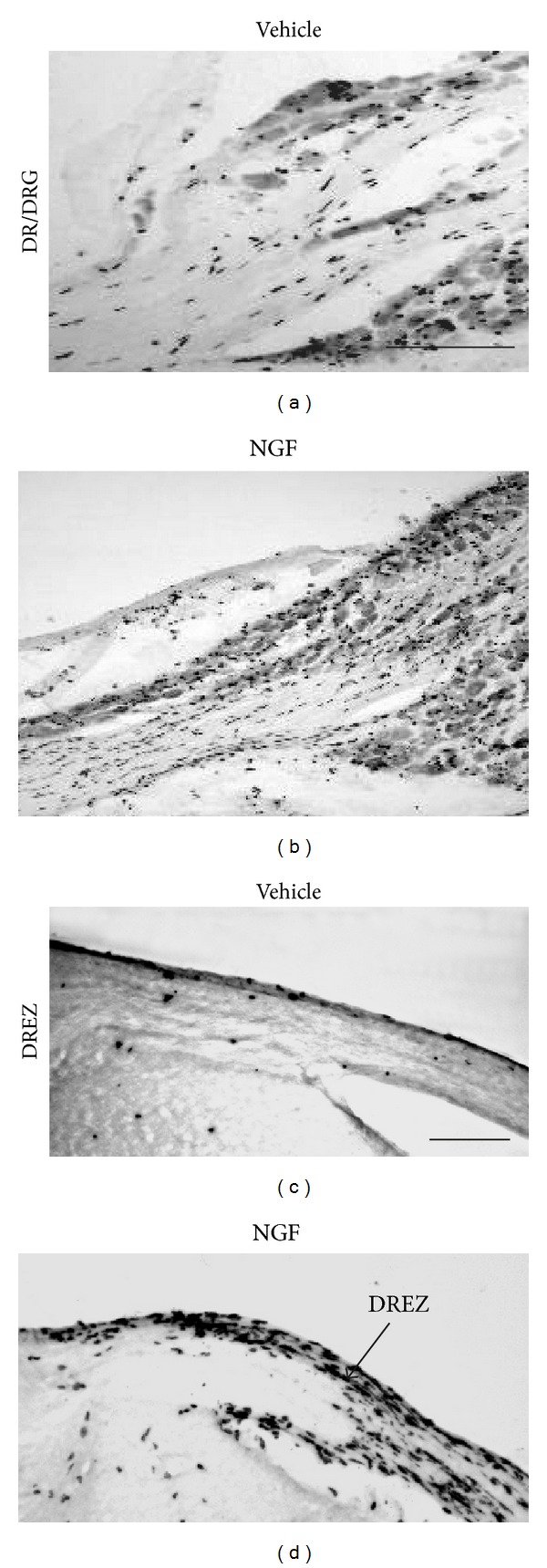
Massive increase of BrdU positive glia along the dorsal root, DRGs, and within the DREZ. Representative BrdU DAB images from vehicle control ((a), (c)) and NGF treated mice ((b), (d)) at the level of the dorsal root (DR) and DRG as well as DREZ ((c), (d)). Scale bar = 200 *μ*m.

To label newly generated cells within the DRG, all animals were injected each day with a single dose of BrdU (Figure 1(b)). Control animals had very few BrdU positive cells in the dorsal root and within the DREZ (Figures 2(a) and 2(c)). In contrast, to the controls, we observed a profound increase of BrdU positive cells in the NGF treated group confined to the dorsal root and the subpial space surrounding the convexly-shaped DREZ at the cervical level (Figures 2(b) and 2(d)). However, we observed only a few BrdU positive cells within the CNS portion of the DREZ and the dorsal column of the spinal cord.

### 3.2. NGF Increases Cell Proliferation within the DRGs

**Figure 3:**
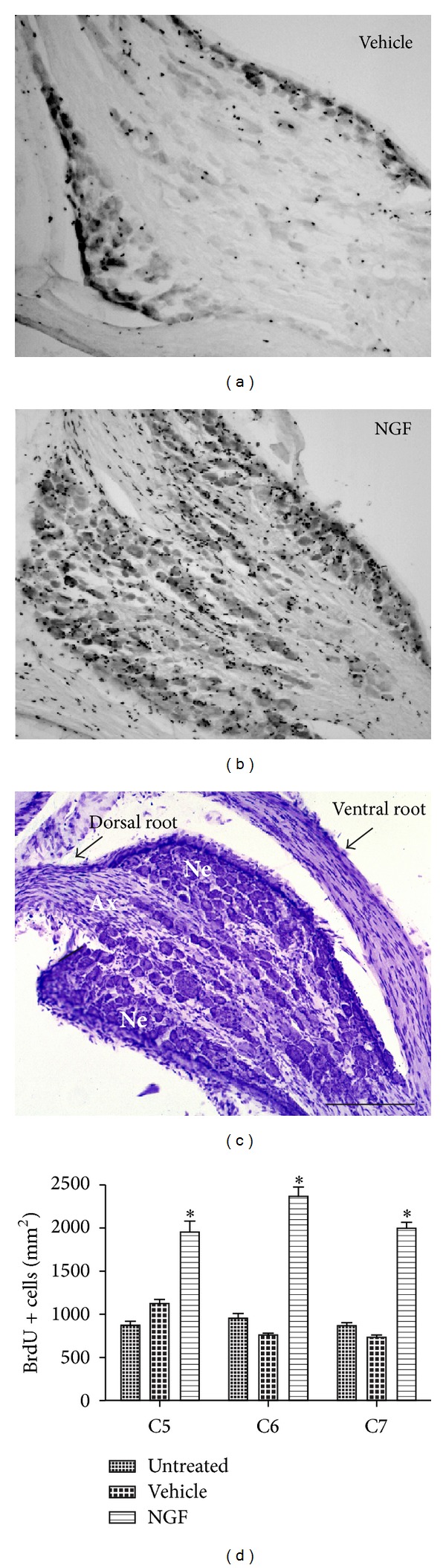
NGF induces a significant increase in the number of proliferating cells within the cervical DRG. Representative BrdU-DAB stainings of cervical DRGs of both the vehicle control (a) and NGF-treated animal (b). Note the massively increased BrdU positive profiles present in the neuronal soma compartment of the NGF-treated animals. Nissl staining of a cervical DRG (c) showing the axonal (Ax) and neuronal soma compartment (Ne). Neuronal soma compartments within the DRG were chosen for quantification of BrdU positive nuclei. Quantification of BrdU positive cells within the neuronal soma compartment across cervical ganglia C5 to C7 (d). Mean ± S.E.M.; *P < 0.01. Scale bar = 200 *μ*m.

ICV infusion of NGF resulted in a massively increased BrdU-labeling in the axonal as well as neuronal soma compartment of the cervical ganglia (Figures 3(a) and 3(b)). The distribution of BrdU positive cells within the neuronal soma compartment of NGF infused animals was evenly distributed among small, medium, and large perikarya. Next, we determined the number of proliferating cells within the neuronal soma of DRGs at cervical segments C5 to C7 (Figure 3(c)). Quantification of the newly generated cells across the cervical DRGs (C5, C6, and C7) revealed that NGF-infused animals showed ~2.5 times more BrdU positive cells per area in the neuronal soma compartment compared to both control groups (Figure 3(d)). There was a significant group difference at each cervical segment analyzed (C5: F_2,6_ = 10.3, *P* < 0.01; C6: F_2,6_ = 207.8, *P* < 0.001; C7: F_2,6_ = 32.3, *P* < 0.001). For the analyzed cervical segments combined, there was a significant treatment effect (F_2,6_ = 97.8; *P* < 0.001). NGF-infusion over two weeks resulted in a labeling of 2120 ± 65 BrdU cells/mm^2^, whereas vehicle treated animals had a mean of 847 ± 28 BrdU positive cells and did not differ from untreated animals (mean: 877 ± 29 BrdU positive cells; *P* > 0.05).

### 3.3. Phenotypic Analysis of Proliferating DRG Cells

**Figure 4:**
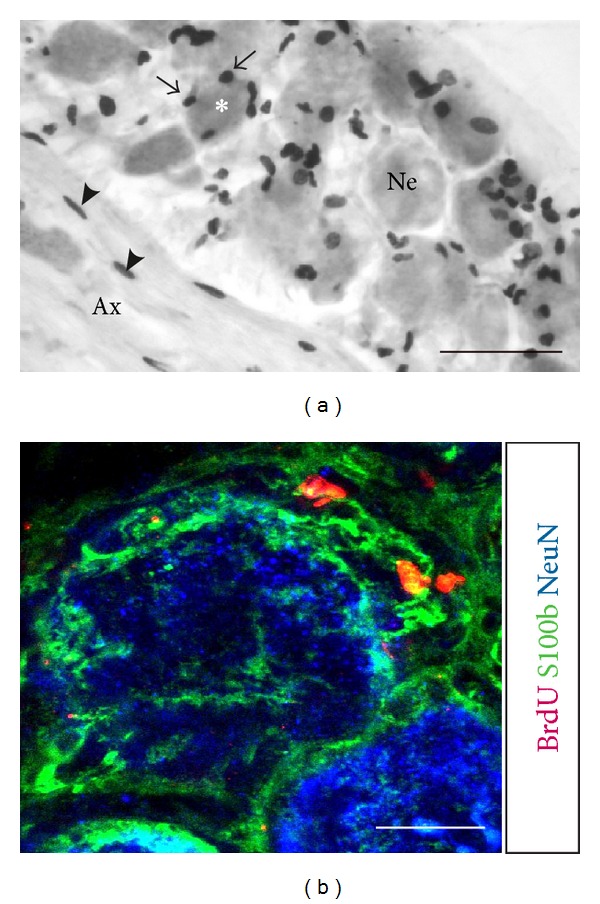
Phenotypic analysis of the proliferating cells within the sensory ganglia. The asterisk marks the perikaryon of a sensory neuron within the neuronal soma compartment (Ne). BrdU positive nuclei, here marked by black arrows, surround the perikaryon. On the lower left side, the axonal compartment (Ax) of the DRG is depicted. Black arrowheads point at elongated BrdU positive nuclei, which are oriented in parallel to axons (a). Scale bar = 50 *μ*m. BrdU positive nuclei (red) are associated with the Schwann and satellite cell marker S100*β* (green) and do not colocalize with NeuN (blue), a marker for neuronal nuclei (b). Scale bar = 20 *μ*m.

BrdU positive nuclei were subdivided into two different populations based on their distinct morphologies: (1) cigar-like nuclei oriented parallel to axonal profiles (Figure 4(a); axonal compartment) and (2) ovoid-shaped nuclei juxtaposed to the perikarya of the sensory ganglion cells (Figure 4(a); neuronal soma compartment). These BrdU cell populations are assumed to represent Schwann and satellite cells, respectively. In order to characterize the phenotype of the proliferating cells in the sensory ganglia we performed triple label immunofluorescence using antibodies against a neuronal marker (NeuN), a glial marker (S100*β*), and BrdU. BrdU positive nuclei were closely juxtaposed to the NeuN positive neuronal nuclei and had an S100*β*-immunoreactive cytoplasm (Figure 4(b)). In addition, S100*β* immunoreactive cells were closely adjacent to the neuronal perikarya without being BrdU positive indicating that a restricted number of glial cells underwent cell division during the two-week infusion period. Since there were no sensory neurons immunoreactive for BrdU, our findings suggest that only glial cells of the DRG undergo mitosis.

### 3.4. NGF Mediated Increase of Glial Proliferation across the Neuraxis

**Figure 5:**
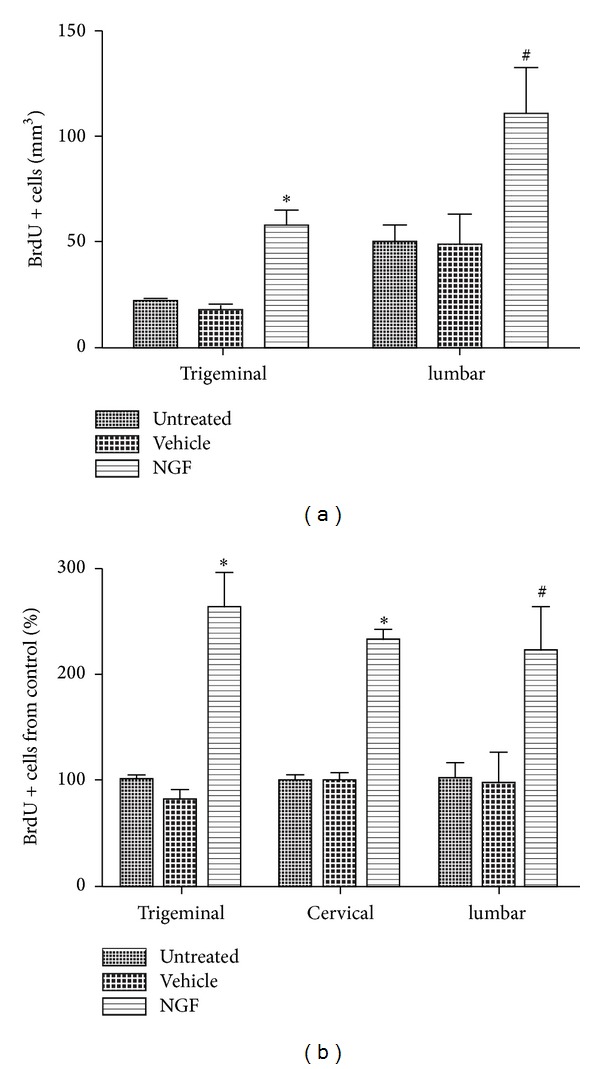
ICV delivery of NGF induces glial proliferation along the neuraxis. Quantification of BrdU positive nuclei within the neuronal soma compartment of trigeminal ganglia and along the lumbar DRGs from levels L2 to L4 (a). The relative magnitude of the NGF-dependent response decreased along the rostral-caudal gradient of the neuraxis (b). Mean ± S.E.M.; *P < 0.01; ^#^ *P* = 0.05.

Unbiased stereological assessment of the trigeminal ganglia revealed also a significant group effect (F_2,6_ = 23.1; *P* < 0.01) after NGF exposure resulting in a more than 3-fold increase of BrdU positive cells compared to vehicle controls (Figure 5(a)). At the lumbar level, the number of BrdU positive cells in the NGF group was still increased by 2.3-fold compared to vehicle controls (F_2,6_ = 4.9; *P* = 0.05; Figure 5(a)). NGF infusion resulted in a labeling of 110 ± 22 BrdU positive cells per mm^3^, whereas vehicle treated animals and untreated animals showed a significant reduction in proliferating cells with a mean of 48 ± 15 and 50 ± 8 BrdU positive cells per mm^3^, respectively. However, NGF induced proliferation of glial cells showed a tendency to decrease along the rostral-caudal gradient of the neuraxis (Figure 5(b)). Compared to trigeminal ganglia, we detected a decrease in BrdU positive cells by 11.3 ± 4.3% and 15.6 ± 16.6% at the level of cervical and lumbar DRGs, respectively.

## 4. Discussion

This study was designed to detect neuronal and glial proliferation in the PNS after exogenous application of NGF via continuous ICV administration. It was hypothesized that NGF exposure throughout the subarachnoid space leads to proliferation of Schwann cells within the dorsal root and DREZ, since previous studies with long-term infusion of NGF showed massive Schwann cell hyperplasia surrounding the medulla and dorsal circumference of the spinal cord in rat and primates [19, 31–33, 39]. In the untreated and vehicle controls we observed scattered BrdU positive cells in all structures analyzed confirming that, physiologically, peripheral glial cells have a low rate of proliferation [40]. However, NGF infusions resulted in a massive increase in BrdU positive cells not only at the DREZ and dorsal root but also within the DRGs. In particular, we stereologically quantified the neuronal soma compartment of the sensory ganglia where we observed a 2.3- to 3-fold increase of BrdU positive cells along the neuraxis.

The present anatomical and phenotypic analysis indicates that NGF induces proliferation of both peripheral glial cells, namely, satellite and Schwann cells. In the axonal compartment of the DRG, along the dorsal root, and within the DREZ, proliferating cells exhibited a more elongated appearance, resembling nuclei of Schwann cells. In the neuronal soma compartment of the DRG, proliferating nuclei around the sensory neurons showed a round or ovoid shape, similar to satellite cells. Using multilabel immunofluorescence, proliferating ovoid S100*β* positive cells were frequently closely juxtaposed to neuronal profiles consistent with proliferating satellite cells.

Satellite and Schwann cells derive from NCSCs, which express p75NTR. During development, neuroblasts initially outnumber glial cells, but this ratio is reversed as satellite cells proliferate to surround the individual ganglionic cell bodies. The proliferation and differentiation of both cell populations, the neuroblasts and glial precursors, may involve the neurotrophins acting via p75NTR. In fact, p75NTR is a strong marker for PNS and neural crest derived progenies and demarcates the DREZ [16, 41]. There are several potential explanations of the observed NGF-mediated mitotic effect on peripheral glial cells. (1) Glial progenitor cells exist within the DRG and give rise to both glial phenotypes. NCSCs in the PNS have been isolated and characterized from adult trigeminal ganglia, dorsal root ganglia, and myenteric ganglia [42–46]. In DRG explants from adult DRG, a cell population was isolated and characterized, showing stem-cell-like properties [43]. These cells were GFAP positive, expressing p75NTR. (2) Schwann cell precursors proliferate within the axonal compartment and along the nerve roots. Furthermore, peripheral nerves may harbor a progenitor niche containing Schwann cell precursors. Finally, Schwann cells have the potential to reenter the cell cycle upon nerve injury [47]. (3) A common progenitor proliferates within sensory ganglia, migrates along the centrally projecting axon into the dorsal root, and does not penetrate into the CNS portion of the transitional zone. Boundary cap cells may constitute this late-preserved reservoir of neural precursors in the PNS [48]. Boundary cap cell progeny has the capacity to migrate along the spinal nerve into the DRG. Moreover, they were able to proliferate and to differentiate into both neuronal and glial cells [48]. However, we did not detect ganglion cells immunoreactive for BrdU. Due to our labeling paradigm, there is still the possibility that neurogenesis exists in the adult PNS since a low rate of neuronal birth or a protracted maturation process may escape detection [49].

To decipher the specific effect of NGF on different glial cells, the DREZ is of particular interest. At the PNS-CNS interface, different cell types, namely, Schwann cells and oligodendrocytes, provide peripheral and central myelination, respectively. We could clearly delineate the topographical specificity of the NGF-dependent response on peripheral glial cells since the convexly shaped central projection into the dorsal root had very few BrdU positive profiles. In contrast, numerous dividing cells were evident within the PNS portion of the transitional zone. This PNS-CNS demarcation of NGF-dependent proliferation is mirrored by a similar spatial p75NTR expression in the PNS, but not in the CNS portion of the transitional zone [50].

Different members of the neurotrophin family are known to promote axonal sprouting and induce hypertrophy of sensory ganglia. These effects depend upon the selective binding of the distinct neurotrophin to its corresponding trk receptor. To date this is the first* in vivo *evidence that systemic administration of neurotrophins has the ability to modulate glial cell turnover in the PNS. This NGF-mediated mitotic effect on satellite and Schwann cells may depend on activation of the p75NTR/trkA receptor system since Schwann and satellite cells express p75NTR [7]. Alternatively, the effect on proliferation may be indirectly mediated via diffusible neuronal- or axonal-associated signals. Since NGF has no mitotic effect on Schwann or satellite cells* in vitro*, one could favor the hypothesis that numerous growth factors present in the axonal and neuronal micromilieu may provide the proliferative signal to both glial populations. Neuregulin, a soluble factor, is secreted by PNS neurons and was shown to be pivotal for the survival and proliferation of the Schwann cell lineage [51–53]. Specifically, neuregulin type III-ß3 stimulates Schwann cell proliferation [54].

In summary, NGF is a potent trophic factor to induce gliogenesis in the PNS when administered continuously into the intrathecal space. However, the mechanisms and the specific cell type linked to this proliferative response remain to be determined.

## 5. Conclusions

We show that NGF administration strongly induces gliogenesis in sensory ganglia, along the dorsal root, and within the DREZ along the neuraxis. Whether this NGF-dependent effect on glial cell proliferation is mediated directly on peripheral glial cells or indirectly via diffusible neuronal—or axonal—associated signals remains to be determined. Our findings indicate that glial cell proliferation within the PNS may play an important role in augmentation of peripheral regeneration within the PNS. Although NGF has the potential to be effective in the treatment of neurodegenerative diseases, for example, Alzheimer's disease or glaucoma, glial proliferation within the PNS has to be considered as a potential adverse effect.
